# Zinc Oxide Nanoparticles Alleviate Chilling Stress in Rice (*Oryza Sativa* L.) by Regulating Antioxidative System and Chilling Response Transcription Factors

**DOI:** 10.3390/molecules26082196

**Published:** 2021-04-11

**Authors:** Yue Song, Meng Jiang, Huali Zhang, Ruiqing Li

**Affiliations:** 1 College of Agronomy, Anhui Agricultural University, Hefei 230036, China; yuesong@zju.edu.cn; 2 National Key Laboratory of Rice Biology, Institute of Crop Sciences, Zhejiang University, Hangzhou 310029, China; mengjiang@zju.edu.cn; 3 State Key Laboratory of Rice Biology, Chinese National Center for Rice Improvement, China National Rice Research Institute, Hangzhou 310029, China; zhanghuali621@126.com

**Keywords:** zinc oxide nanoparticles, chilling stress, rice, chilling response, antioxidative system

## Abstract

As one of the common abiotic stresses, chilling stress has negative effects on rice growth and development. Minimization of these adverse effects through various ways is vital for the productivity of rice. Nanoparticles (NPs) serve as one of the effective alleviation methods against abiotic stresses. In our research, zinc oxide (ZnO) NPs were utilized as foliar sprays on rice leaves to explore the mechanism underlying the effect of NPs against the negative impact of chilling stress on rice seedlings. We revealed that foliar application of ZnO NPs significantly alleviated chilling stress in hydroponically grown rice seedlings, including improved plant height, root length, and dry biomass. Besides, ZnO NPs also restored chlorophyll accumulation and significantly ameliorated chilling-induced oxidative stress with reduced levels of H_2_O_2_, MDA, proline, and increased activities of major antioxidative enzymes, superoxide dismutase (SOD), catalase (CAT), and peroxidase (POD). We further found that foliar application of ZnO NPs induced the chilling-induced gene expression of the antioxidative system (*OsCu/ZnSOD1*, *OsCu/ZnSOD2*, *OsCu/ZnSOD3*, *OsPRX11*, *OsPRX65*, *OsPRX89*, *OsCATA*, and *OsCATB*) and chilling response transcription factors (*OsbZIP52*, *OsMYB4*, *OsMYB30*, *OsNAC5*, *OsWRKY76*, and *OsWRKY94*) in leaves of chilling-treated seedlings. Taken together, our results suggest that foliar application of ZnO NPs could alleviate chilling stress in rice via the mediation of the antioxidative system and chilling response transcription factors.

## 1. Introduction

Rice (*Oryza sativa* L.), a major crop in the world and the staple food for over half of the world’s population, is mostly a tropical crop, but its distribution also extends to temperate and subtropical regions [1,2]. However, rice usually encounters environmental stresses such as extreme temperatures, drought, flooding, and high salinity [3], which greatly influence the growth and development of rice. Temperature is one of the vital factors for the production of rice, as the ideal temperature for rice growth ranges from 25 to 30 °C [4]. As previously reported, the growth and germination of the rice plant is negatively influenced when the temperature of the environment declines below 15 °C, and chilling stress that occurs below 20 °C in the stages of grain-filling, booting, and flowering would result in an impossibility to grow rice on about 7 × 10^6^ hectares of land in Southeast and South Asia [5,6,7]. Therefore, discovering the underlying mechanisms of the chilling response in rice will offer a theoretical basis for breeding chilling-tolerant varieties.

Chilling stress adversely affects the metabolic and physiological functions of rice, and thus reduces the yield [8,9]. The effects of chilling stress on rice growth and development involve different physiological pathways: affecting photosynthesis through the inhibition of chloroplast formation and chlorophyll biosynthesis in rice leaves [10]; inducing the accumulation of signal substances, e.g., malondialdehyde (MDA) and reactive oxygen species (ROS) [11,12]; accumulating compatible osmolytes, e.g., free proline [13] and impacting antioxidants, such as superoxide dismutase (SOD), catalase (CAT), and peroxidase (POD) [14,15].

Besides, there are numerous transcription factors involved in the chilling response and tolerance of rice, including bZIP (basic leucine zipper), MYB (v-myb avian myeloblastosis viral oncogene homolog), NAC (NAM, ATAF, and CUC), and WRKY (tryptophan-arginine-lysine-tyrosine) [16]. A recent study revealed that one bZIP transcription factor, OsbZIP52, suppresses chilling resistance by inhibiting chilling-stress-related genes (e.g., *OsTPP1*) [16]. Multiple studies reported that MYB transcription factors play a vital role in chilling tolerance. For instance, the R2R3-type OsMYB4 transcription factor regulates the rice chilling stress response independently via *OsDREBs* [17,18]. OsMYB30 inhibits the chilling tolerance by down-regulating the gene expression of *β-AMYLASE* (*BMY*) via interaction with JASMONATE ZIM-DOMAIN 9 (OsJAZ9) [19]. Moreover, a NAC transcription factor encoding gene, OsNAC5, enhances the tolerance of transgenic rice seedlings to chilling stress [20]. Furthermore, WRKY transcription factors, OsWRKY76 and OsWRKY94, confer chilling tolerance [21,22].

Minimization of chilling stress through various ways is vital for the productivity of rice. Nanoparticles (NPs) serve as an effective alleviation method against abiotic stresses. Advances in nanomaterials (NMs) can rise the production of crops in the present opposing environment. Present studies have shown that nanotechnology is able to enhance the development and growth of crops and alleviate the negative effects of abiotic stress [23]. Mohammadi et al. found that the application of TiO_2_ NPs not only alleviated the membrane damage under cold stress but also prevented oxidative stress in chickpeas [24]. TiO_2_ treatment also increased the crop tolerance to cold stress via maintaining the stability of carotenoid and chlorophyll accumulations, inducing the activity of superoxide dismutase, ascorbate peroxidase, and catalase [25], and enhancing the gene expression of chlorophyll- and rubisco-binding proteins [26].

Nonetheless, previous studies have shown that ZnO NPs could alleviate abiotic stress (e.g., toxicity of cadmium) in plants [27], while the function of ZnO NPs in mitigating chilling stress is largely unknown. For this reason, we sprayed ZnO NPs to observe the effect of ZnO NPs on alleviating chilling stress in this research. Finally, we found that foliar application of ZnO NPs mitigated the chilling stress in hydroponically grown rice seedlings with induced chlorophyll accumulation and reduced oxidative stress. Potential underlying mechanisms of ZnO NPs on alleviation of chilling stress are also discussed.

## 2. Results

### 2.1. Details of ZnO NPs

**Figure 1:**
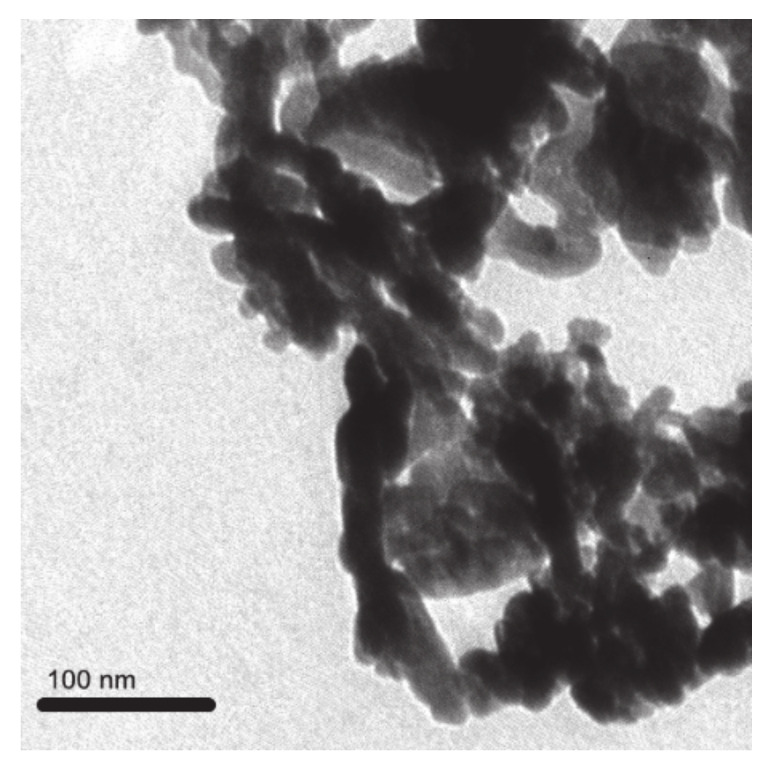
Characterization of the zinc oxide nanoparticles (ZnO NPs). Transmission electron microscopy (TEM) images of the ZnO NPs. Scale bars, 100 nm.

The NPs of ZnO were purchased from Chaowei Nano Technology Co., Ltd. (Shanghai, China). The particle morphology and size distribution of ZnO NPs were observed by transmission electron microscopy (TEM; Tecnai F20 S-TWIN, FEI Co., Hillsboro, OR, USA). The complete details of the ZnO NPs are shown in Table S1. The TEM images (Figure 1) revealed that the ZnO NPs were spherical with regular diameters of 30 nm. The size distribution of the ZnO NPs in media (50 mg/L TX-10) with a concentration of 100 mg/L was determined with a Malvern Zetasizer (Nano ZS, Malvern, UK). To minimize agglomeration, we sonicated the different concentrations of ZnO NPs suspensions for 1 h before use.

### 2.2. ZnO NPs Significantly Mitigated Chilling Stress in Rice

**Figure 2:**
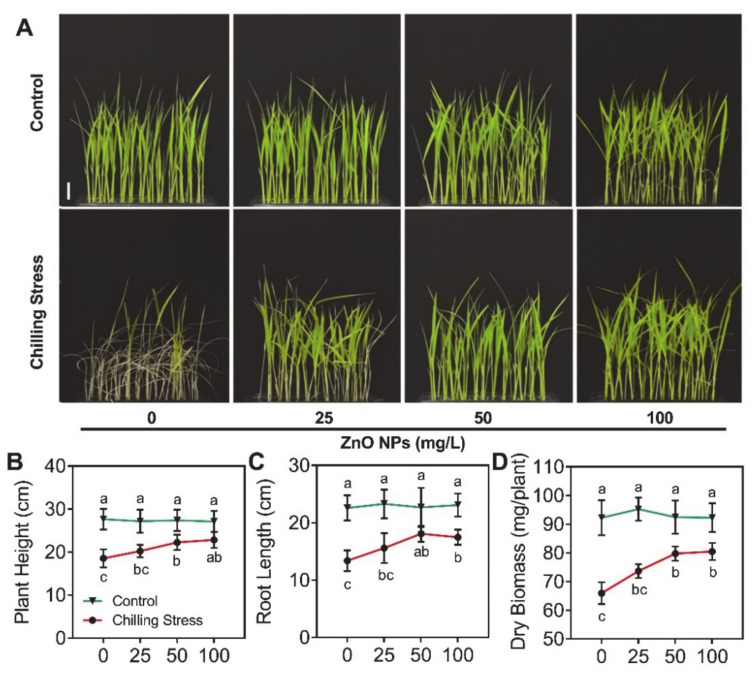
ZnO NPs alleviate chilling stress in rice. Phenotype (**A**), plant height (**B**), root length (**C**), and dry biomass (**D**) of rice seedlings subjected to 0, 25, 50, and 100 mg/L of ZnO NPs under chilling stress were measured, respectively. The tests were repeated six times. The data given are the averages of six replicates, with the standard deviation (SD) shown by the error bars. Different letters above or below error bars show the differences at *p* < 0.05. Scale bar = 5 cm (**A**).

**Figure 3:**
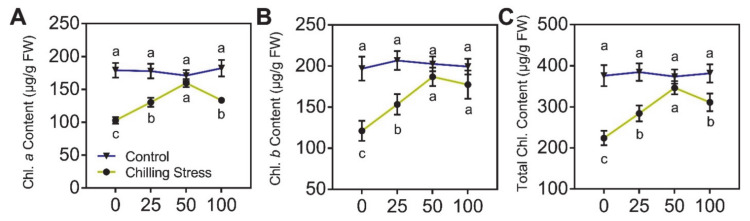
Effects of ZnO NPs on the content of chlorophyll (Chl.) in rice seedlings under chilling stress. Chl. *a* (**A**), Chl. *b* (**B**), and total Chl. (**C**) in leaves of rice seedlings subjected to 0, 25, 50, and 100 mg/L of ZnO NPs under chilling stress were measured, respectively. The tests were repeated three times. The data given are the averages of three replicates, with the standard deviation (SD) shown by the error bars. Different letters above or below error bars show the differences at *p* < 0.05. FW, fresh weight.

To evaluate the effect of ZnO NPs application on chilling stress, 20-day-old rice plants were exposed to the chilling condition (16 h light/8 h dark, at 10 °C). The low temperature (10 °C) caused toxicity in rice seedlings (Figure 2A). After 5 days of chilling stress treatment and 10 days of recovery, the plant height, root length, and dry biomass significantly decreased by 32.9%, 40.7%, and 28.5%, respectively compared with those of untreated control (Figure 2B–D). Rice seedlings also exhibited visible chlorosis in young leaves, and the content of total chlorophyll dramatically decreased by 40.4% after chilling stress treatment (Figure 3), compared to untreated control.

**Figure 4:**
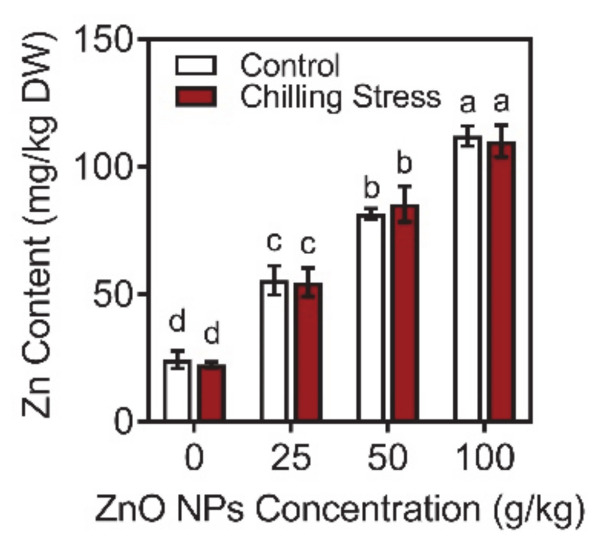
Zn accumulation in rice leaves. Zn accumulation of rice seedlings subjected to 0, 25, 50, and 100 mg/L of ZnO NPs under chilling stress were measured, respectively. The tests were repeated three times. The data given are the averages of three replicates, with the SD shown by the error bars. Different letters above error bars show the differences at *p* < 0.05. DW, dry weight.

In contrast, the inhibition of growth of rice seedlings under chilling stress was alleviated after foliar application of ZnO NPs with different concentrations. The content of the Zn element in rice leaves was analyzed (Figure 4) after removal of ZnO NPs, which were physically adsorbed on the surfaces of leaves using a KI/I_2_ solution. After the application of 25, 50, and 100 mg/L ZnO NPs, the Zn content in leaves dramatically increased, namely 42.5-, 20.3-, and 45.0-fold, compared to the application of 0 mg/L ZnO NPs under chilling stress (Figure 4). Similar results were displayed without chilling stress (Figure 4). The supplementation with ZnO NPs displayed a good performance in the alleviation of chilling stress in rice seedlings, which was reflected in a significantly higher value of plant height, root length, and dry biomass of rice plants. Moreover, by spraying with different concentrations of ZnO NPs, the symptoms of chlorosis in rice leaves were significantly relieved and the accumulation of total chlorophyll was partially (25 mg/L) or totally (50 and 100 mg/L) restored (Figure 3).

### 2.3. Application of ZnO NPs Alleviated Chilling-Induced Oxidative Stress

**Figure 5:**
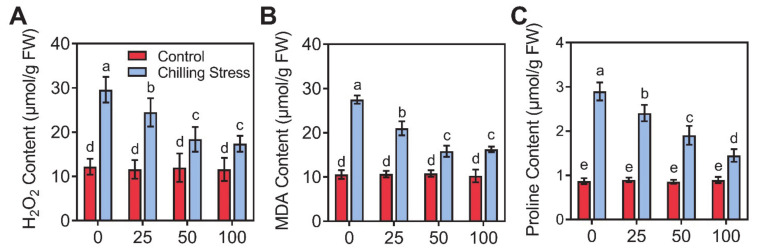
Effects of ZnO NPs on oxidative damage in rice leaves under chilling stress. Hydrogen peroxide (H_2_O_2_, **A**), malondialdehyde (MDA, **B**), and proline (**C**) of rice seedlings subjected to 0, 25, 50, and 100 mg/L of ZnO NPs under chilling stress were measured, respectively. The tests were repeated three times. The data given are the averages of three replicates, with the SD shown by the error bars. Different letters above error bars show the differences at *p* < 0.05. FW, fresh weight.

**Figure 6:**
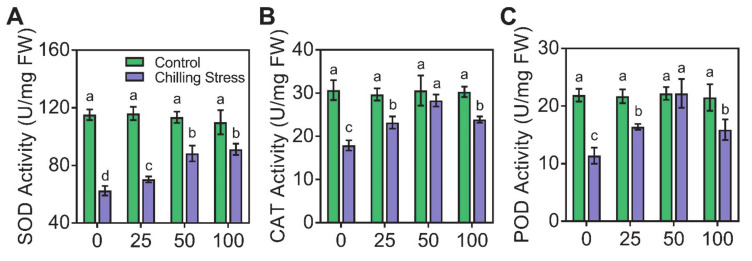
Effects of ZnO NPs on antioxidant enzyme activity in rice leaves under chilling stress. Superoxide dismutase (SOD, **A**), catalase (CAT, **B**), and peroxidase (POD, **C**) of rice seedlings subjected to 0, 25, 50, and 100 mg/L of ZnO NPs under chilling stress were measured, respectively. The tests were repeated three times. The data given are the averages of three replicates, with the SD shown by the error bars. Different letters above error bars show the differences at *p* < 0.05. FW, fresh weight.

To further understand how ZnO NPs alleviated the chilling stress, we measured the contents of H_2_O_2_, MDA, and proline, and the activities of antioxidant enzymes (SOD, CAT, and POD) in rice plants (Figure 5 and Figure 6). On one hand, we found that chilling stress caused severe oxidative stress, as indicated by the increased ROS in non-radical form H_2_O_2_ (Figure 5A), which was severely toxic and caused serious damage to the plant cells. Chilling stress treatment strongly increased the levels of H_2_O_2_, MDA, and proline in rice leaves by 2.42-, 2.60-, and 3.33-fold, respectively, compared to control (Figure 5). The supplementation of 100 mg/L ZnO NPs significantly reduced H_2_O_2_, MDA, and proline contents in leaves by 41.2%, 40.9%, and 50.0%, respectively, compared to single chilling stress (Figure 5). On the other hand, we found that chilling stress greatly reduced the antioxidant enzymes (SOD, CAT, and POD) activity in leaves by 45.8%, 41.6%, and 47.7%, respectively, compared to control (Figure 6). The application of 100 mg/L ZnO NPs greatly increased the activities of SOD, CAT, and POD in chilling-treated rice seedlings compared to those under only chilling stress (Figure 6).

**Figure 7:**
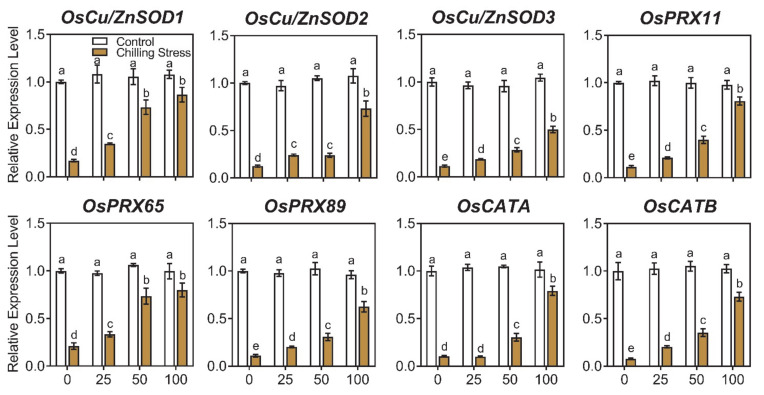
Effects of ZnO NPs on gene expression of antioxidant enzymes in rice leaves under chilling stress. Gene expression of rice seedlings subjected to 0, 25, 50, and 100 mg/L of ZnO NPs under chilling stress was measured, respectively. Gene expression was first normalized to the *UBQ5* gene in rice as an internal control and executed relative to each gene’s expression of control (assigned a value of 1). The data given are the averages of three replicates, with the SD shown by the error bars. Different letters above error bars show the differences at *p* < 0.05.

To investigate the molecular basis of how ZnO NPs reduces oxidative stress in rice, the gene expression of antioxidant enzymes containing *OsCu/ZnSOD1*, *OsCu/ZnSOD2*, *OsCu/ZnSOD3*, *OsPRX11*, *OsPRX65*, *OsPRX89*, *OsCATA*, and *OsCATB* was measured. Chilling stress significantly decreased the gene expression of antioxidant enzymes (SOD, CAT, and POD) compared to control (Figure 7 and Figure S1), while the application of different concentrations of ZnO NPs greatly increased the gene expression of SOD, CAT, and POD, in chilling-treated rice plants compared to only chilling stress (Figure 7 and Figure S1). These results indicated that ZnO NPs application could relieve oxidative stress in rice seedlings exposed to chilling stress by reducing ROS and enhancing the expression of antioxidant enzymes.

### 2.4. ZnO NPs Reduced Gene Expression of Chilling Response Transcription Factors under Chilling Stress in Rice

**Figure 8:**
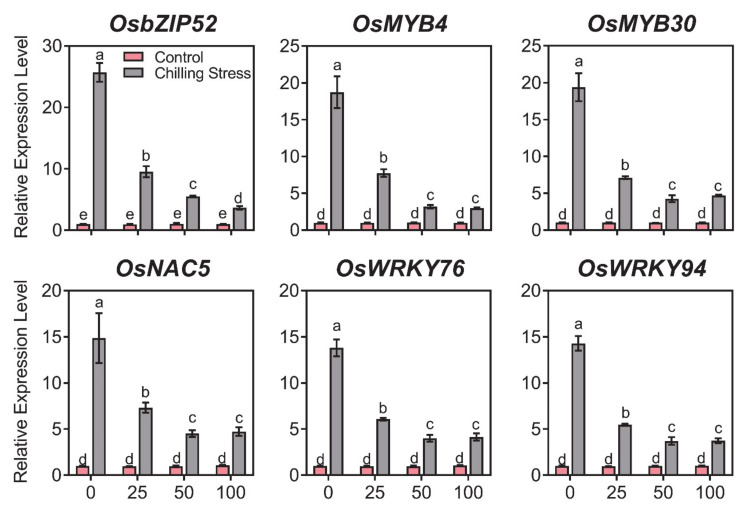
Effects of ZnO NPs on gene expression of chilling response transcription factors in rice leaves under chilling stress. Gene expression of rice seedlings subjected to 0, 25, 50, and 100 mg/L of ZnO NPs under chilling stress was measured, respectively. Gene expression was first normalized to the *UBQ5* gene in rice as an internal control and executed relative to each gene’s expression of control (assigned a value of 1). The data given are the averages of three replicates, with the SD shown by the error bars. Different letters above error bars show the differences at *p* < 0.05.

To investigate the molecular basis of how ZnO NPs alleviates chilling stress in rice, the genes of chilling response transcription factors including *OsbZIP52*, *OsMYB4*, *OsMYB30*, *OsNAC5*, *OsWRKY76*, and *OsWRKY94* were chosen for gene expression analysis in rice leaves (Figure 8 and Figure S2). Exposure to chilling stress significantly induced the expression of *OsbZIP52*, *OsMYB4*, *OsMYB30*, *OsNAC5*, *OsWRKY76*, and *OsWRKY94* in rice leaves. Application of ZnO NPs restored the expression of all above-mentioned genes to control level after chilling stress in rice leaves (Figure 8 and Figure S2). These results demonstrated that the reduced gene expression of the chilling response may be an important mechanism for the ZnO NPs-produced alleviation.

## 3. Discussion

Applications of ZnO NPs to resistance to stresses (drought stress, salinity stress, cadmium and lead toxicity) have been reported in several plants. The supplementation of ZnO NPs increased the seed germination percentage and rate, and decreased the seed fresh and dry weight of soybean (*Glycine max* L.) under drought stress [28]. Besides, under salinity stress, feeding with ZnO NPs also greatly improve plant growth and development in sunflower (*Helianthus annuus* L.) [29] and wheat (*Triticum aestivum* L.) [30]. Also, ZnO NPs alleviated cadmium (Cd) toxicity in wheat by enhancing wheat growth and Zn concentrations, while reducing the Cd concentration in plants [27]. ZnO NPs alleviated the bioavailability of cadmium and lead and changed the uptake of iron in hydroponically grown lettuce (*Lactuca sativa* L.) [31]. However, no related research has reported the effects of ZnO NPs on rice seedlings under chilling stress. In this study, we found that foliar application of ZnO NPs efficiently mitigated chilling stress in rice seedlings. In particular, ZnO NPs application significantly increased the resistance to chilling stress in rice by restoring chlorophyll biosynthesis, reducing the level of ROS, enhancing the activity of antioxidant enzymes, and regulating the gene expression of chilling stress response transcription factors.

Firstly, to confirm that the ZnO NPs were absorbed by rice plants, we measured the content of Zn element in rice leaves with different treatments (Figure 4). Consistent with previous reports on plants [32,33], our results revealed that the accumulation of ZnO NPs greatly increased under both control and chilling stress conditions in rice seedlings with different supplements of ZnO NPs (Figure 4), while there was no difference in the content of Zn element between control and chilling stress treatment under different supplements of ZnO NPs (Figure 4). These results demonstrated that with the increase in the amount of ZnO NPs sprayed, the Zn concentration in the organs of rice leaves increased but was not affected by chilling stress.

Chilling stress adversely affects the metabolic and physiological functions of rice, and thus reduces the yield [8,9,34,35]. Chilling stress impacts rice growth and development by affecting photosynthesis, with the inhibition of chloroplast formation and chlorophyll biosynthesis in rice leaves [10,36,37]. Similar to previous studies, rice seedlings also exhibited visible chlorosis in young leaves, and the content of total chlorophyll (chlorophyll *a* and *b*) was significantly reduced after the chilling stress treatment (Figure 3) compared to untreated control. Furthermore, by spraying with ZnO NPs, the symptoms of chlorosis in rice leaves were significantly relieved, and the accumulation of total chlorophyll was restored (Figure 3).

The oxidative bursts produced by chilling stress can induce the accumulation of signal substances, e.g., malondialdehyde (MDA) and reactive oxygen species (ROS) [11,12]; accumulate compatible osmolytes, e.g., free proline [13] and impact antioxidants, such as superoxide dismutase (SOD), catalase (CAT), and peroxidase (POD) [14,15]. However, the responses change in different higher plants and display dose-, tissue-, development-, and genotype-dependency [38]. The activity of CAT in rice markedly decreased, and the contents of H_2_O_2_ and MDA greatly increased, by chilling stress treatment compared to control [15]. The activity of SOD and CAT in some varieties of rice seedlings displayed small changes due to chilling stress, and other varieties of rice showed greatly reduced levels; however, the contents of H_2_O_2_ and MDA increased under chilling stress in most tested varieties of rice [39]. In our research, the activity of all of the antioxidant enzymes measured reduced after chilling stress (Figure 6), which was similar to the reports of Sohag et al. [15] and Wang et al. [39]. The gene expression of antioxidant enzymes containing *OsCu/ZnSOD1*, *OsCu/ZnSOD2*, *OsCu/ZnSOD3*, *OsPRX11*, *OsPRX65*, *OsPRX89*, *OsCATA*, and *OsCATB* was also induced with the application of ZnO NPs under chilling stress (Figure 7). Reduced SOD, CAT, and POD activity and gene expression might lead to an oxidative burst after chilling stress in rice seedlings, as revealed by the ROS level, such as H_2_O_2_ in chilling-treated rice, and finally caused induced lipid peroxidation, e.g., MDA (Figure 5, Figure 6 and Figure 7). Foliar application of ZnO NPs alleviated chilling-produced oxidative stress by enhancing antioxidant enzyme activity and reducing ROS content in rice seedlings. The oxidative stress was greatly mitigated by ZnO NPs, demonstrating that improving the antioxidant defense capacity may be an important mechanism for the ZnO NPs-produced alleviation.

Different transcription factors involved in the chilling tolerance and chilling response in rice, namely *OsbZIP52* [16], *OsMYB4* [17,18], *OsMYB30* [19], *OsNAC5* [20], *OsWRKY76* [21], and *OsWRKY94* [22], were reported to be greatly induced under chilling stress in related studies. In this study, all the above chilling response genes tested increased under chilling stress (Figure 8), which was similar to the former reports [16,17,18,19,20,21,22]. ZnO NPs reduced the expression of these chilling-stress-induced response genes, thereby alleviating various toxicities caused by chilling stress. Foliar application of ZnO NPs decreased the gene expression of the chilling response in rice seedlings, which demonstrated that the reduced gene expression of the chilling response may be an important mechanism for the ZnO NPs-produced alleviation.

In conclusion, we demonstrated that foliar application of ZnO NPs could efficiently mitigate the toxicity of chilling stress in rice. In particular, ZnO NPs significantly reduced the toxicity of chilling stress in rice leaves by regulating the gene expression of chilling stress response transcription factors, indicating ZnO NPs played a vital role in regulating the chilling response. ZnO NPs also had influential and multiple effects on plant growth and chlorophyll biosynthesis, finally enhancing the antioxidant potential and the abilities of ROS scavenging under chilling stress. Therefore, our findings not only illustrate the effect and mechanism of ZnO NPs in suppressing the toxicity of chilling stress in rice, but also provide a practicable solution for reducing chilling stress effects on crops.

## 4. Materials and Methods

### 4.1. Plant Materials and Treatments

The seeds of rice (*Oryza sativa* ssp Japonica cultivar Nipponbare) were sterilized with 20% (*v*/*v*) NaClO and then soaked in ultrapure water at 30 °C for 2 days. A total of 48 rice seeds were then transplanted into 96-well plastic hydroponic boxes including 1/2 Murashige-Skoog (MS) standard liquid medium, and planted in a growth chamber with 75% relative humidity (normal condition: 16 h light at 30 °C/8 h dark at 25 °C). For the foliage ZnO NPs spraying, 10-day-old rice plants grown in diverse boxes including 1/2 MS standard liquid medium under the normal condition were sprayed 50 mL ZnO NPs solution (25, 50, and 100 mg/L ZnO NPs with 50 mg/L TX-10) daily for 10 days. The 50 mg/L TX-10 replicates were utilized as controls. For the chilling stress treatment, 20-day-old rice plants were placed under the chilling condition (16 h light/8 h dark, at 10 °C) for 5 days. Then, the chilling-treated rice seedlings were moved to the normal condition for 1 week. The 32-day-old rice seedlings with or without ZnO NPs treatment were sampled for phenotypical, physiological, and molecular detections. Six replicates (the replicates here correspond to one plant) were tested for the measurements of plant height, root length, and dry biomass, and three replicates (the replicates here correspond to three to six plants depending on the weight) were detected for other molecular, physiological, and biochemical measurements.

### 4.2. Measurement of Zinc (Zn) Content

The concentration of Zn in rice leaves was measured according to a previous method [40]. Briefly, Zn absorbed on the surface of seedlings was eliminated by immersing the root in 20 mM Na_2_-EDTA (disodium ethylenediamine tetra-acetic acid) for 60 min, and then rinsing five times with distilled water. The sample of rice was dried at 105 °C for 120 min and at 60 °C for 48 h. The weight of whole seedlings was measured as dry biomass after reaching an unchanging weight. Dried plant samples (about 0.1 g) were mixed with 6 mL of HNO_3_ and kept in the reaction with a Multiwave (MARS 6, CEM Co., Matthews, NC, USA) at 140 °C for about 2 h. Finally, the content of Zn was measured with the atomic absorption spectrometer AA-7000 (Shimadzu, Tokyo, Japan).

### 4.3. Determination of Chlorophyll (Chl.)

The content of Chl. in rice leaves was quantified as previously reported [41]. About 100 mg of fresh rice leaves were mixed and ground with 10 mL 80% (*v*/*v*) acetone. After being kept in darkness for 2 h, the mixture was centrifuged at 12,000× *g* for 10 min. Finally, the absorbance at 663 and 645 nm was measured, respectively.

### 4.4. Hydrogen Peroxide (H_2_O_2_) Detection

The H_2_O_2_ content was measured using a hydrogen peroxide assay kit (Solarbio, Beijing, China) [42]. The content of H_2_O_2_ in rice leaves was detected by measuring the absorbance of titanium-peroxide complex formation at 415 nm.

### 4.5. Measurement of Malondialdehyde (MDA) Content

The accumulation of MDA was determined following a previously described protocol [43]. Approximately 100 mg of rice leaves were homogenized and mixed with 10 mL 10% (*v*/*v*) of trichloroacetic acid, and the mixture was then centrifuged at top speed (12,000× *g*) for 20 min. The supernatant was mixed with an equal volume of thiobarbituric acid and then kept at 95 °C for 30 min. The mixture was centrifuged at 12,000× *g* for 30 min after being quickly cooled on ice, and the supernatant absorbance at 450, 532, and 600 nm was measured, respectively.

### 4.6. Measurement of Free Proline (Pro)

The content of free proline was measured as previously reported [44]. About 1 g of rice leaves were ground with an aqueous solution of 3% (*v*/*v*) sulfosalicylic acid. After centrifugation at room temperature at 12,000× *g* for 15 min, 2 mL of supernatant was added to the same volumes of glacial acetic acid and acidic ninhydrin. This mixture was heated at 95 °C for 40 min, cooled on ice, and then followed by the addition of 4 mL of toluene to extract the colored reaction product separated from the aqueous phase. The absorbance of the toluene phase at 520 nm was determined, and the standard curve of proline was utilized to assay the accumulation of free proline.

### 4.7. Antioxidant Enzyme Activities Determination

The activity of the antioxidant enzymes containing superoxide dismutase (SOD), catalase (CAT), and peroxidase (POD) was measured using respective commercial kits (Solarbio, Beijing, China) as previously published [45,46,47]. The standardization of antioxidant enzymes activity was described: one unit of SOD activity was expressed as the suppression of photoreduction of 4-nitro blue tetrazolium chloride (NBT) by half under light; one unit of CAT activity was detected according to the decomposition of 1 nmol H_2_O_2_ per min; one unit of POD activity was assayed by determining an absorbance (470 nm) increase of 0.01 per min.

### 4.8. Gene Expression Assay

Relative gene expression was determined as previously published [48,49]. The RNAprep Pure Plant Kit (Tiangen, Beijing, China) and HiScript III 1st Strand cDNA Synthesis Kit (Vazyme Biotech Co., Ltd., Nanjing, China) were used to extract and reverse-transcribe the total RNA from rice leaves. AceQ qPCR SYBR Green Master Mix (Vazyme, Nanjing, China) was used to apply the quantitative real-time PCR (qRT-PCR). The qRT-PCR primers are listed in Table S2. The ACTIN gene in rice was used as an internal control (Table S2), and the level of gene expression was measured following the method of 2^−ΔΔCt^ [50].

### 4.9. Statistical Analyses

The data in the figures and tables is presented as means with standard deviation (SD) because there were six or three replications. The mean differences were assessed using ANOVA. The statistical significance of differences between treatments was assessed using Bonferroni post-hoc tests.

## Data Availability

The data presented in this study are available in the article or Supplementary Materials.

## Supplementary Materials

The following are available online. Figure S1: Expression profiles of antioxidant enzymes genes in rice leaves, Figure S2: Expression profiles of chilling response transcription factors genes in rice leaves, Table S1: Characterization details of ZnO NPs, Table S2: Primers used in the present study.

## Sample Availability

Samples of the compounds are not available from the authors.
