# Supplementary material for: Zinc Oxide Nanoparticles Alleviate Chilling Stress in Rice (Oryza Sativa L.) by Regulating Antioxidative System and Chilling Response Transcription Factors
Source: Molecules. 2021 Apr 11;26(8):2196. doi: 10.3390/molecules26082196 (PMC8069548; doi:10.3390/molecules26082196)
Supplement: Supplementary file 1 [file molecules-26-02196-s001.pdf]

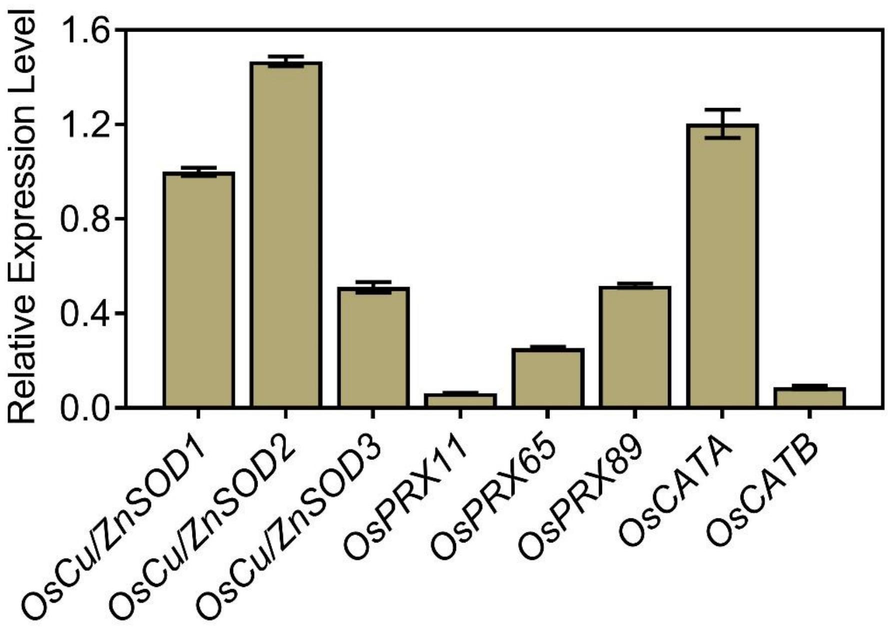

Figure S1. Expression profiles of antioxidant enzymes genes in rice leaves. The expression levels were first normalized to the rice internal control gene UBQ5 and reported relative to OsCu/ZnSOD1 expression level of control (assigned a value of 1). All analyses were performed with three replicates.

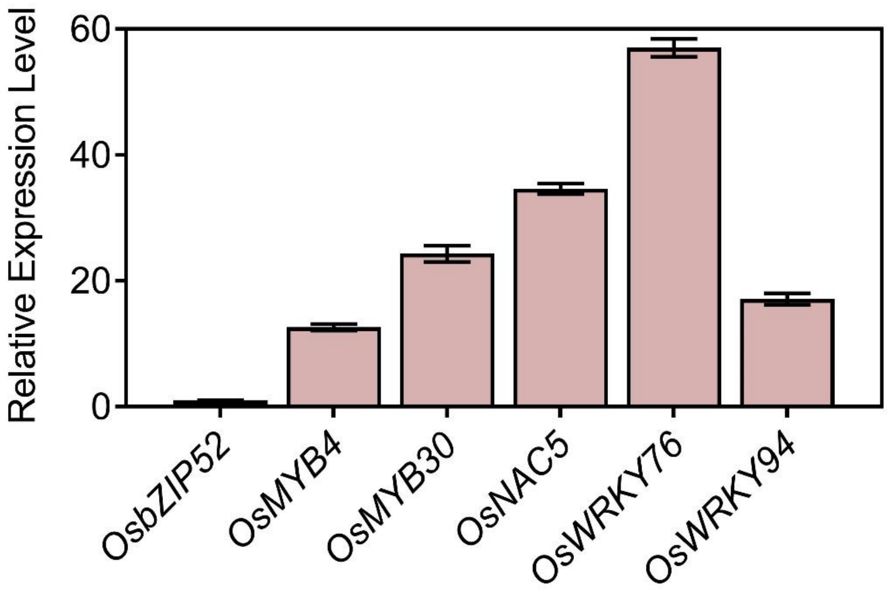

Figure S2. Expression profiles of chilling response transcription factors genes in rice leaves. The expression levels were first normalized to the rice internal control gene *UBQ5* and reported relative to *OsCu/ZnSOD1* expression level of control (assigned a value of 1). All analyses were performed with three replicates.

Table S1. Characterization details of ZnO NPs.

NPs	Size<br>nm	Zeta potential<br>mV	Mobility<br>μmcm/Vs	Conductivity<br>mS/cm
ZnO	30	6.697	0.525	0.010

Table S2. Primers used in the present study.

Gene Name	LOC No.	Forwad Primer Sequence (5'→3')	Reverse Primer Sequence (5'→3')
UBQ5	*Os06g0650100*	CCGACTACAACATCCAGAAGGAG	AACAGGAGCCTACGCCTAAGC
OsCu/ZnSOD1	*Os03g0351500*	ATCCTGATGATCTTGGAAAGGG	AAAACACATAGTTCATTGGGCG
OsCu/ZnSOD2	*Os07g0665200*	CTTAGCAAGACCACTGGAAATG	TGTTGGAAAGTTGAGACGTCTA
OsCu/ZnSOD3	*Os08g0561700*	CACCCTTTTTGTCATCTTCGAG	TTACTGTGAGACGAACGATCAA
OsPRX11	*Os01g0294700*	TACCGCGACCTCCTCAACTA	GAAGCCCGGCATGAACCT
OsPRX65	*Os05g0134400*	CTCCCGGACTCCACCTTCA	GGAGCAGTGGCCGACG
OsPRX89	*Os06g0681600*	TTCCACGACTGCTTCGTCAG	TCTCGACCACGCTCTTGATG
OsCATA	*Os02g0115700*	TCCCAGTGTGATGAGTCGTTGG	TCTTCACATGCTTGGCTTCACG
OsCATB	*Os06g0727200*	TCCTACTGGTCGCAGTGTGATG	TTTCAGGTTGAGACGTGAAGCC
OsbZIP52	*Os06g0662200*	CTGTTAGAAGCAGAGAGGAGTC	TTCAGATAACTGTGCCTGCTTT
OsMYB4	*Os04g0517100*	GTGAAGCAAAGCTAAAATGGGT	GGGGGATCAGATCAGATAAGTC
OsMYB30	*Os02g0624300*	CTTCTGGCTCAAGCTGTTCATC	AATGAACAAGAGATCGACCCAA
OsNAC5	*Os11g0184900*	AGGTGGACCTCTACAAGTTCAA	CGAGAAGAAGTACCACTCCTTC
OsWRKY76	*Os09g0417600*	TTTGATTACTCGAGCTCCAGAG	CATTGGTCATCGACTTGGAATC
OsWRKY94	*Os12g0597700*	AGGAGGAAGTTGCGAATTAAGA	CGCTGCTACATTTGTAGTAACC
